# *Acinetobacter seifertii* Isolated from China: Genomic Sequence and Molecular Epidemiology Analyses

**DOI:** 10.1097/MD.0000000000002937

**Published:** 2016-03-07

**Authors:** Yunxing Yang, Jianfeng Wang, Ying Fu, Zhi Ruan, Yunsong Yu

**Affiliations:** From the Department of Infectious Diseases, Sir Run Run Shaw Hospital, School of Medicine, Zhejiang University, Hangzhou, Zhejiang, China.

## Abstract

Clinical infections caused by *Acinetobacter* spp. have increasing public health concerns because of their global occurrence and ability to acquire multidrug resistance. *Acinetobacter calcoaceticus*–*Acinetobacter baumannii* (ACB) complex encompasses *A. calcoaceticus*, *A. baumannii*, *A. pittii* (formerly genomic species 3), and *A nosocomial* (formerly genomic species 13TU), which are predominantly responsible for clinical pathogenesis in the *Acinetobacter* genus.

In our previous study, a putative novel species isolated from 385 non-*A. baumannii* spp. strains based on the *rpoB* gene phylogenetic tree was reported. Here, the putative novel species was identified as *A. seifertii* based on the whole-genome phylogenetic tree. *A. seifertii* was recognized as a novel member of the ACB complex and close to *A. baumannii* and *A. nosocomials.* Furthermore, we studied the characteristics of 10 *A. seifertii* isolates, which were distributed widely in 6 provinces in China and mainly caused infections in the elderly or children. To define the taxonomic status and characteristics, the biochemical reactions, antimicrobial susceptibility testing, pulsed field gel electrophoresis (PFGE), multilocus sequence typing (MLST), and whole-genome sequence analysis were performed.

The phenotypic characteristics failed to distinguish *A. serfertii* from other species in the ACB complex. Most of the *A. seifertii* isolates were susceptible to antibiotics commonly used for nosocomial *Acinetobacter* spp. infections, but one isolate (strain A362) was resistant to ampicillin/sulbactam, ceftazidime and amikacin. The different patterns of MLST and PFGE suggested that the 10 isolates were not identical and lacked clonal relatedness.

Our study reported for the first time the molecular epidemiological and genomic features of widely disseminated *A. seifertii* in China. These observations could enrich the knowledge of infections caused by non-*A. baumannii* and may provide a scientific basis for future clinical treatment.

## INTRODUCTION

The genus *Acinetobacter* is widely distributed in nature and commonly occurs in soil. During the past decades, it has been increasingly recognized as a significant pathogen of nosocomial infections, including ventilator-associated pneumonia, bloodstream infections, skin and soft-tissue infections, meningitis, and urinary tract infections.^1–3^ Most of the studies of the etiological organisms concentrate on *A. baumannii*, which is notorious for its multidrug resistance or even pan-drug resistance.^2,4,5^ The insufficient knowledge in databases and the intrinsic intragenus similarity make current phenotypic tests difficult to distinguish different *Acinetobacter* species, especially between members of the *Acinetobacter calcoaceticus*–*Acinetobacter baumannii* (ACB) complex (including *A. baumannii*, *A. calcoaceticus*, *A. nosocomials,* and *A. pittii*).^6,7^*A. seifertii* was recognized as a novel member of the ACB complex by Nemec et al.^8^ It was formerly known as gen sp “close to 13TU,”^9^ which was isolated from human clinical specimens and the environment in different countries and areas. Further, study of *A. seifertii* is necessarily required.

In this study, we report on the detection of *A. seifertii* in China, mainly using the whole-genome sequence and molecular typing methods to clarify the phylogenetic relationships with other *Acinetobacter* species and molecular epidemiology characteristics.

## MATERIALS AND METHODS

### Bacterial Strains and Phenotypic Characteristics

In our previous study,^10^ we reported a putative, novel *Acinetobacter* species: A total of 385 non-*A. baumannii* isolates were collected from 27 provinces in China from January 2009 to September 2010.^11^ By 16S rRNA and RNA polymerase β-subunit gene (*rpoB*) sequencing, we found that the most common species was *A. pittii* (49.09%).^10^ Nevertheless, 10 isolates constituted a novel cluster and could not be assigned into any previously known species (GenBank accession numbers: KF982810-KF982820). Here, we chose the 10 isolates to study further. The colonies were observed after 18 to 24 h at 37°C on tryptic soy agar (Oxoid). Utilization tests were evaluated by VITEK 2 system (Sysmex-bioMérieux, Marcy l’Etoile, France).

### Antimicrobial Susceptibility Testing

The antibiotic susceptibility profile of all isolates to different antibiotics, including ampicillin/sulbactam, ceftazidime, imipenem, colistin, amikacin, tigecycline, tetracycline, ciprofloxacin, aztreonam, and fosfomycin, was determined by the Etest (AB bioMérieux, Solna, Sweden), and the interpretation was according to the CLSI 2015 guidelines.^12^ The breakpoints for Enterobacteriaceae of the European Committee on Antimicrobial Susceptibility Testing were used for tigecycline and aztreonam. (http://www.eucast.org/). *Escherichia coli* ATCC 25922 was used as a reference strain for quality control.

### Pulsed Field Gel Electrophoresis

Genomic DNA was digested by the restriction enzyme ApaI. The conditions were 22 h at 6 V/cm and 14°C, with a pulse angle of 120 degree, and pulse time from 5 to 35 s with a CHEF-Mapper XA pulsed field gel electrophoresis (PFGE) system (Bio-Rad, Hercules, CA, USA). *Salmonella enterica* serotype Braenderup H9812 was used as the size marker.^13^ The restriction patterns were analyzed with BioNumerics 7.0 (Applied Maths BVBA, Sint-Martens-Latem, Belgium). Interpretation was performed according to Tenover's criteria.^14^

### Multilocus Sequence Typing

Multilocus sequence typing (MLST) following the Oxford scheme was performed as described by Bartual et al.^15^ The internal fragments of seven housekeeping genes, including *gltA*, *gyrB*, *gdhB*, *recA*, *cpn60*, *gpi*, and *rpoD*, were PCR-amplified. PCR reactions were designed as follows: predenaturation at 94°C for 5 min, followed by 35 cycles of denaturation at 94°C for 1 min, annealing at 55°C for 30 s, and extension at 72°C for 60 s. Sequence types (STs) were assigned using the PubMLST database (http://pubmlst.org/abaumannii/).

### Whole-Genome Sequence and Phylogenetic Analysis

We chose 3 isolates (strains A354, A360, and A362) for whole-genome sequencing. Total DNA was extracted and sequenced using next-generation sequencing technology (either Illumina HiSeq2000^TM^ with 2 × 100 bp paired-end reads or Illumina MiSeq^TM^ with 2 × 300 bp paired-end reads). The derived short reads were assembled into contigs using CLC Genomics Workbench 8.0 (CLC bio, Denmark). Acquired resistance genes and virulence genes were screened using the ResFinder 2.1 tool on the CGE server (https://cge.cbs.dtu.dk/services/ResFinder/).

Similarities of protein-coding sequences were determined using the BLASTP program of the NCBI Basic Local Alignment Search Tool (BLAST). For a coding sequence to be considered homologous, the protein identity had to be >80%, e-value smaller than 1e^−10^, and aligned length >80% of the gene sequence. Phylogenetic reconstruction was performed using the core genes were shared by the genome of the compared *Acinetobacter* spp. strains with the MEGA 5.0 Maximum-likelihood program and BacWGSTdb platform.^16,17^ The similarity of protein-encoding genes and average amino acid among *A. seifertii*, *A. baumannii*, and *A. nosocomials* was converted to a Venn diagram using R (http://www.r-project.org/), which shows the number of the genes in the specific strains.

### Nucleotide Sequence Accession Numbers

The nucleotide sequence data reported here have been submitted to the GenBank database with the assigned accession number: LFZQ01 (strain A354), LFZR01 (strain A360), and LFZS01 (strain A362).

## RESULTS AND DISCUSSION

### Whole-Genome Sequence Analyses

According to our previous work, there were 10 isolates clustered in the same branch in the *rpoB*-based phylogenetic tree among a total of 385 non-*A. baumannii* isolates.^10^ The *rpoB* gene sequence of the 10 isolates were the closest matched with *A. seifertii*, indicating they probably belong to this species. Thus, the 3 isolates (strains A354, A360, and A362) were chosen for whole-genome sequencing.

**FIGURE 1:**
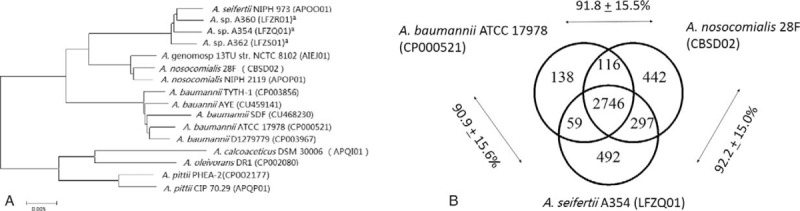
Phylogenetic relationship between 3 strains of putative novel species and other *Acinetobacter* spp. (A) Whole-genome phylogenetic tree of the 3 strains and other sequenced *Acinetobacter* spp. genomes. ^a^Indicates the 3 strains that were chosen for whole-genome sequencing in this study. (B) Genome comparison between *A. seifertii*, *A. baumannii*, and *A. nosocomials.* The numbers in the Venn diagram represent the shared genes between the compared strains. Data outside the Venn diagram represent average amino acid identity between the adjacent strains ± standard deviation.

Genome comparison revealed that a total of 1941 core genes were shared by the genome of the compared *Acinetobacter* spp. strains (Figure 1 A). The phylogenetic tree based on the shared core genes showed that the 3 isolates (strain A354, A360, and A362) constituted the same branch with *A. seifertii* NIPH 973^T^ (formally *Acinetobacter* gen. sp. “close to 13TU”), which is relatively closer to *A. baumannii* and *A. nosocomials* but distant from *A. calcoaceticus* (Figure 1A). These 3 isolates were clustered with each other and constituted a cohesive group. According to the whole-genome-based phylogenetic tree, we concluded that these isolates were identified as *A. seifertii.*

A deeper look inside the ACB complex showed that *A. seifertii* was highly similar to *A. baumannii* and *A. nosocomials*. For example, 83.01% of protein-encoding genes of *A. seifertii* were shared with *A. baumannii* and *A. nosocomials*, with the average amino acid identity over 90% (Figure 1B). The genomes of A354 and A360 isolates both contained only one antimicrobial resistance gene *bla*_ADC-25_. In contrast, A362 contained much more resistance genes, such as *sul2*, *aph(3’)-Via*, *sul1*, *bla*_PER-1_, *aacA4*, *aac(6’)Ib-cr*, *msr(E)*, *mph(E)*, *aac(3)-IId*, *floR*, and *ARR-3*. This probably explains why this isolate exhibits a much higher MICs of ampicillin/sulbactam, ceftazidime, amikacin, aztreonam, and fosfomycin than other isolates. Most of these resistance genes were also commonly present in multidrug-resistant *A. baumannii*. Thus, it is reasonable to hypothesize that *A. seifertii* and *A. baumannii* share the common repertoire of resistance genes to survive in the nosocomial environment.^18^ All 3 isolates carried most of *A. baumannii* known virulence genes, including *ompA*, *pgaABCD*, *csu pili*, *lpsB*, *pmrB*, *pbpG*, *eps*, and *ptk*; so the pathogenicity of *A. seifertii* is likely equal to *A. baumannii*.

### Epidemiological and Clinical Features

Ten *A. seifertii* isolates were distributed in 6 provinces in China, which are geographically distant places. The patients were mostly the elderly and children in several wards. The clinical information of 10 *A. seifertii* isolates is shown in Table 1.

**TABLE 1:**
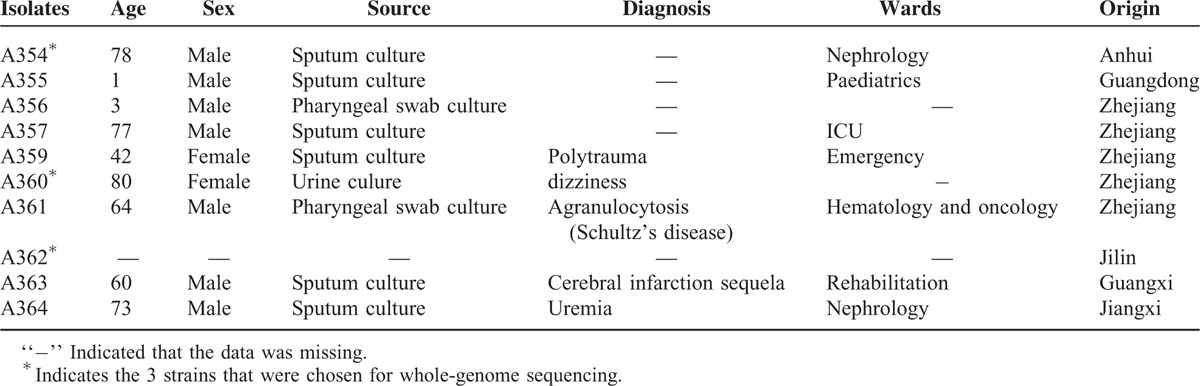
The Clinical Features of 10 *A. seifertii* Isolates

### Phenotypic Characteristics

The phenotypic characteristics of *A. seifertii* were not significantly different from other *Acinetobacter* species, especially the ACB complex. The colonies were 1 to 1.5 mm in diameter, circular, convex, smooth, and slightly opaque with entire margins. Growth occurred in brain-heart infusion (Oxoid) at temperatures ranging from 15°C to 41°C, and the optimum temperature is 37°C. The optimum pH and NaCl concentration was 5.5 to 9 and 0 to 4%, respectively. The isolates of *A. seifertii* were Gram-negative, strictly aerobic, oxidase-negative, catalase-positive, and nonmotile coccobacilli. Overall, *A. seifertii* cannot be reliably distinguished from the ACB complex merely based on phenotypic tests, and therefore it should be a member of the ACB complex; the deposited strain is A354 (=CGMCC 1.15326 = KCTC 42723).

### Antimicrobial Susceptibility Testing

The antibiotic resistance profiles of *A. seifertii* were determined (Table 2). They were susceptible to ciprofloxacin, imipenem, tigecycline, and colistin. All but one isolate showed resistance to fosfomycin (256 ≥ 1024 mg/mL) and aztreonam (32 ≥ 256 mg/mL). Only one isolate (strain A362) was resistant to ampicillin/sulbactam, ceftazidime, and amikacin, and two isolates were resistant to tetracycline.

**TABLE 2:**
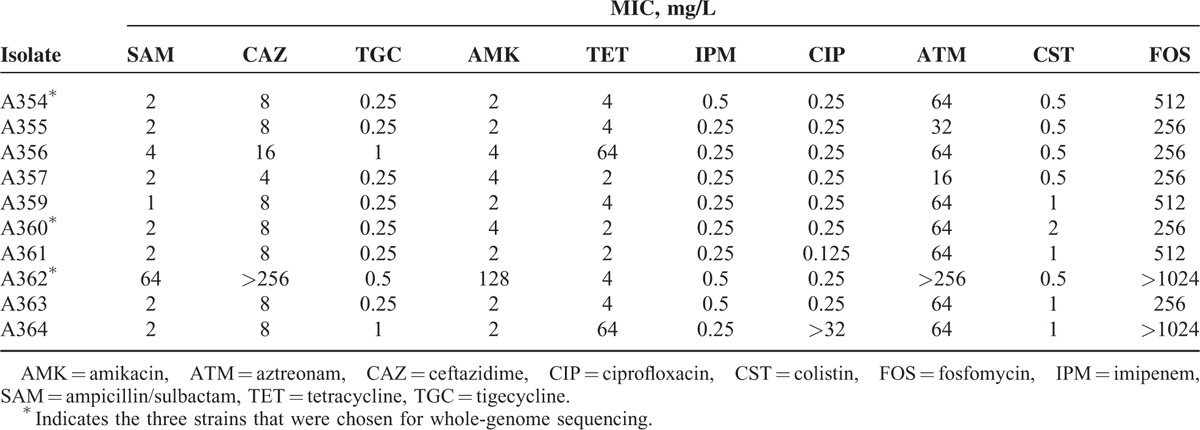
The Minimal Inhibitory Concentration (MIC) of 10 *A. seifertii* Isolates

### Molecular Epidemiology Characteristics

**FIGURE 2:**
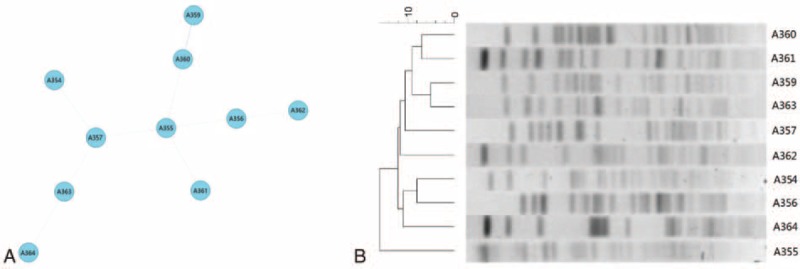
Molecular epidemiology characteristics of 10 *A. seifertii* isolates. (A) Minimum spanning tree analysis of 10 *A. seifertii* isolates based on multilocus sequence typing data. Each circle represents independent sequence type (ST). The lines connecting the circles indicate the relationship between different STs. Different types of lines represent a difference in 3 alleles (dashed lines) and ≥4 alleles (dotted lines). (B) Pulsed field gel electrophoresis analysis for the 10 *A. seifertii* isolates (variation within 3 bands indicates the same clone).

We further identified intra-species genetic diversity by MLST. The 10 isolates exhibited 10 different allele combinations (Figure 2A), and none of them differed from other isolates by <2 alleles. PFGE was also performed. The PFGE profiles presented that 10 isolates were not identical and lacked clonal relatedness (Figure 2B). These typing results were consistent with the clinical information that the 10 isolates were collected from different areas; it also suggested that *A. seifertii* was a widely distributed species, rather than through population movement with an identical clone.

In conclusion, the putative novel species in our previous study was identified as *A. seifertii*. The whole-genome sequence analyses represented that *A. seifertii* shares some common resistant genes with *A. baumannii* to benefit their survival in the nosocomial environment, and the mechanisms of acquiring resistant genes need further study. The clinical information and molecular epidemiology analyses highlighted that *A. seifertii* was distributed geographically with different clones. The further study of *A. seifertii* could enrich the knowledge of infection by non-*A. baumannii* and provide a scientific basis for future clinical treatment. In addition, detailed virulence and epidemiology of *A. seifertii* require further investigation.

## References

[1] Peleg AY Seifert H Paterson DL Acinetobacter baumannii: emergence of a successful pathogen. *Clin Microbiol Rev* 2008; 21:538–582. 18625687 10.1128/CMR.00058-07 PMC2493088

[2] Dijkshoorn L Nemec A Seifert H An increasing threat in hospitals: multidrug-resistant Acinetobacter baumannii. *Nat Rev Microbiol* 2007; 5:939–951. 18007677 10.1038/nrmicro1789

[3] Bergogne-Berezin E Towner KJ Acinetobacter spp. as nosocomial pathogens: microbiological, clinical, and epidemiological features. *Clin Microbiol Rev* 1996; 9:148–165. 8964033 10.1128/cmr.9.2.148 PMC172888

[4] Biedenbach DJ Bouchillon SK Hoban DJ Antimicrobial susceptibility and extended-spectrum beta-lactamase rates in aerobic gram-negative bacteria causing intra-abdominal infections in Vietnam: report from the Study for Monitoring Antimicrobial Resistance Trends (SMART 2009-2011). *Diagnost Microbiol Infect Dis* 2014; 79:463–467. 10.1016/j.diagmicrobio.2014.05.009 24923210

[5] Kuo SC Chang SC Wang HY Emergence of extensively drug-resistant Acinetobacter baumannii complex over 10 years: nationwide data from the Taiwan Surveillance of Antimicrobial Resistance (TSAR) program. *BMC Infect Dis* 2012; 12:200. 22929085 10.1186/1471-2334-12-200 PMC3462144

[6] Nemec A Krizova L Maixnerova M Genotypic and phenotypic characterization of the Acinetobacter calcoaceticus-Acinetobacter baumannii complex with the proposal of Acinetobacter pittii sp. nov. (formerly Acinetobacter genomic species 3) and Acinetobacter nosocomialis sp. nov. (formerly Acinetobacter genomic species 13TU). *Res Microbiol* 2011; 162:393–404. 21320596 10.1016/j.resmic.2011.02.006

[7] Lee MJ Jang SJ Li XM Comparison of rpoB gene sequencing, 16S rRNA gene sequencing, gyrB multiplex PCR, and the VITEK2 system for identification of Acinetobacter clinical isolates. *Diagnost Microbiol Infect Dis* 2014; 78:29–34. 10.1016/j.diagmicrobio.2013.07.013 24157058

[8] Nemec A Krizova L Maixnerova M Acinetobacter seifertii sp. nov., a member of the Acinetobacter calcoaceticus-Acinetobacter baumannii complex isolated from human clinical specimens. *Int J SystEvol Microbiol* 2015; 65 (Pt 3):934–942. 10.1099/ijs.0.000043 25563912

[9] Gerner-Smidt P Tjernberg I Acinetobacter in Denmark: II. Molecular studies of the Acinetobacter calcoaceticus-Acinetobacter baumannii complex. *APMIS* 1993; 101:826–832. 8286091

[10] Wang J Ruan Z Feng Y Species distribution of clinical Acinetobacter isolates revealed by different identification techniques. *PLoS One* 2014; 9:e104882. 25120020 10.1371/journal.pone.0104882 PMC4132069

[11] Ruan Z Chen Y Jiang Y Wide distribution of CC92 carbapenem-resistant and OXA-23-producing Acinetobacter baumannii in multiple provinces of China. *Int J Antimicrob Agent* 2013; 42:322–328. 10.1016/j.ijantimicag.2013.06.019 23988720

[12] Tan SY Chua SL Liu Y Comparative genomic analysis of rapid evolution of an extreme-drug-resistant Acinetobacter baumannii clone. *Genome Biol Evol* 2013; 5:807–818. 23538992 10.1093/gbe/evt047 PMC3673627

[13] Hunter SB Vauterin P Lambert-Fair MA Establishment of a universal size standard strain for use with the PulseNet standardized pulsed-field gel electrophoresis protocols: converting the national databases to the new size standard. *J Clin Microbiol* 2005; 43:1045–1050. 15750058 10.1128/JCM.43.3.1045-1050.2005 PMC1081233

[14] Tenover FC Arbeit RD Goering RV Interpreting chromosomal DNA restriction patterns produced by pulsed-field gel electrophoresis: criteria for bacterial strain typing. *J Clin Microbiol* 1995; 33:2233–2239. 7494007 10.1128/jcm.33.9.2233-2239.1995 PMC228385

[15] Bartual SG Seifert H Hippler C Development of a multilocus sequence typing scheme for characterization of clinical isolates of Acinetobacter baumannii. *J Clin Microbiol* 2005; 43:4382–4390. 16145081 10.1128/JCM.43.9.4382-4390.2005 PMC1234098

[16] Ruan Z Feng Y BacWGSTdb, a database for genotyping and source tracking bacterial pathogens. *Nucleic Acid Res* 2016; 44 (D1):D682–D687. 26433226 10.1093/nar/gkv1004 PMC4702769

[17] Tamura K Peterson D Peterson N MEGA5: molecular evolutionary genetics analysis using maximum likelihood, evolutionary distance, and maximum parsimony methods. *Mol Biol Evol* 2011; 28:2731–2739. 21546353 10.1093/molbev/msr121 PMC3203626

[18] Poirel L Bonnin RA Nordmann P Genetic basis of antibiotic resistance in pathogenic Acinetobacter species. *IUBMB Life* 2011; 63:1061–1067. 21990280 10.1002/iub.532

